# Botulinum Neurotoxin Injections in Children with Self-Injurious Behaviors

**DOI:** 10.3390/toxins15040236

**Published:** 2023-03-23

**Authors:** Mariam Hull, Mered Parnes, Joseph Jankovic

**Affiliations:** 1 Pediatric Movement Disorders Clinic, Section of Pediatric Neurology and Developmental Neuroscience, Texas Children’s Hospital and Baylor College of Medicine, Houston, TX 77030, USA; 2 Parkinson’s Disease Center and Movement Disorders Clinic, Department of Neurology, Baylor College of Medicine, Houston, TX 77030, USA

**Keywords:** self-injurious behaviors, self-injurious biting, malignant Tourette, botulinum toxin

## Abstract

Self-injurious behaviors are repetitive, persistent actions directed toward one’s body that threaten or cause physical harm. These behaviors are seen within a broad spectrum of neurodevelopmental and neuropsychiatric conditions, often associated with intellectual disability. Injuries can be severe and distressing to patients and caregivers. Furthermore, injuries can be life-threatening. Often, these behaviors are challenging to treat and require a tiered, multimodal approach which may include mechanical/physical restraints, behavioral therapy, pharmacotherapy, or in some cases, surgical management, such as tooth extraction or deep brain stimulation. Here, we describe a series of 17 children who presented to our institution with self-injurious behaviors in whom botulinum neurotoxin injections were found helpful in preventing or lessening self-injury.

## 1. Introduction

Self-injurious behaviors (SIB) are repetitive and persistent actions directed toward one’s body, which threaten or produce significant physical injury without the intention of self-harm [1,2]. These are common in neurodevelopmental and neuropsychiatric conditions, often in the setting of co-occurring intellectual disability or other neurobehavioral disorders, such as Tourette syndrome [1,3]. Prevalence estimates range from 4% in the general population up to 30–50% in certain genetic conditions and intellectual disability [1,4]. The behaviors can be quite variable and have included hitting, biting, picking, head-banging, hair-pulling, scratching, kicking, and cutting, among others (Video S1). In addition to causing patients and caregivers distress, SIB can become disabling and, in some cases, life-threatening [2,4,5,6]. Severe complications have included scarring, retinal detachment, and death [4].

**Figure 1:**
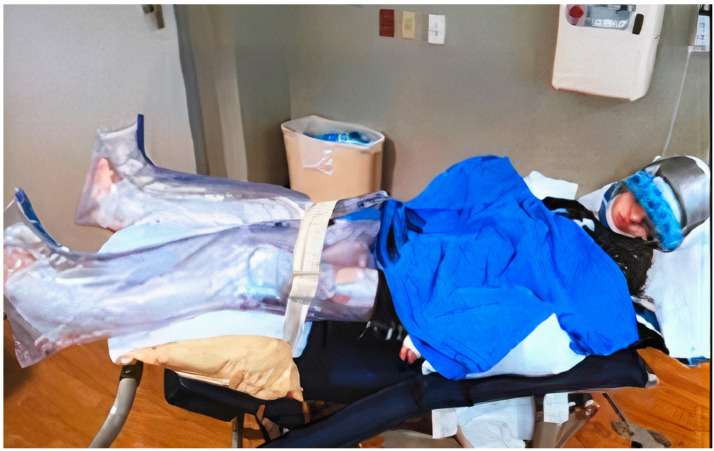
Example of physical restraints some caregivers use to minimize self-injurious behaviors.

Treatment of SIB is complex and requires multiple modalities based on the etiology as well as the type of SIB [7]. Whenever possible, disease-specific treatment is considered first. For example, in the case of Lesch-Nyhan syndrome (LNS), S-adenosylmethionine or probenecid may be used [1]. In most cases, no treatment exists, and symptomatic or supportive treatment is sought. Physical/mechanical restraints are often utilized first and have included protective gloves, arm splints, helmets, and mouth guards (Figure 1). It is important to note, however, that these mechanical restraints can themselves potentially cause harm, such as bruising, lacerations, and aspiration in the case of mouth guards, and must be used with caution in young children. Behavioral therapy is often applied and can be helpful in some patients [1,2,8]. Pharmacotherapy is also frequently used, but its efficacy varies [1,7,8]. Finally, surgical therapy, such as deep brain stimulation, can be considered in severe and refractory cases [1,9]. However, data on the efficacy of these interventions are limited.

Botulinum neurotoxin (BoNT) is used in numerous neurologic and non-neurologic conditions with dozens of indications within the field of movement disorders [10]. Common indications in movement disorders include spasticity, hemifacial spasm, tremor, tics, and dystonia in both children and adults [10,11,12,13,14]. BoNT has also been suggested as helpful in treating migraine as a cause of head-banging, and self-injurious behaviors in children with autism spectrum disorder [15] and has been discussed in the treatment of self-mutilating behaviors in LNS [16].

Here, we describe a series of children, adolescents, and young adults with SIB treated with BoNT injections.

## 2. Results

Seventeen patients (11 male) were evaluated in our pediatric movement disorders clinic, with ages ranging from 11 months to 20 years for management of SIB and treated with BoNT (onabotulinumtoxinA) (Table 1). The most common comorbid diagnoses included intellectual disability/global developmental delay. Etiologies of the intellectual disability/global developmental delay included congenital insensitivity to pain with anhidrosis, cardiofaciocutaneous syndrome, LNS, perinatal tuberculous meningitis with ischemic stroke, carnitine transporter deficiency, suspected mitochondrial disorder, Aicardi syndrome, and three had an unknown cause of intellectual disability (Table 2). Length of follow-up ranged from 10 months to 3 years (average 1.4 years), except for one patient that was lost to follow-up.

**Figure 2:**
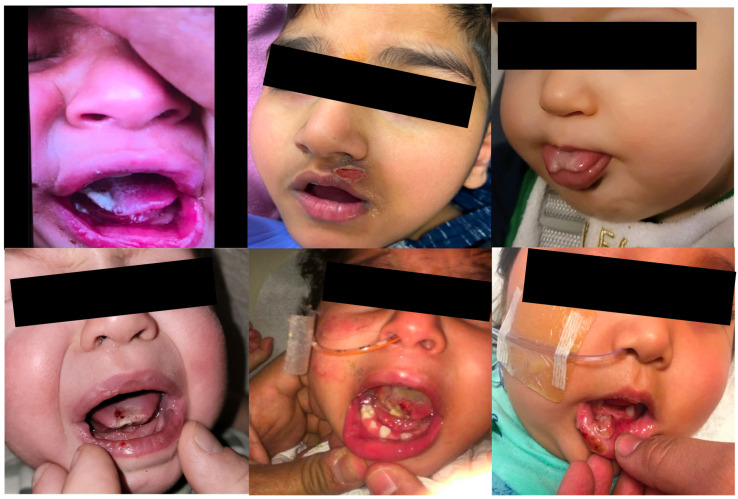
Injuries sustained by patients with self-injurious biting prior to botulinum neurotoxin injections include partial amputation of the tongue, infections, and scarring of the lip and tongue.

**Figure 3:**
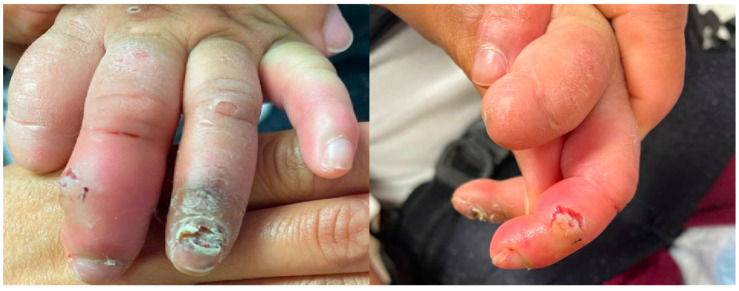
Finger injuries due to self-injurious biting include partial amputation of digits, infection, and skin breakdown.

Fifteen patients had utilized other treatments for SIB, with the most common being physical restraints (often using multiple types) (Table 2). All treatments were ineffective or poorly tolerated due to adverse effects. Two patients had not tried other treatments and had severe and forceful whiplash neck extension tics. Only one patient underwent behavioral modification therapy, which resulted in no improvement of SIB. Sites and dosing of BoNT were chosen based on the involved muscles in the SIB as well as estimated muscle mass and force of muscle contraction (Table 3 and Table 4) [17,18,19]. All patients who received BoNT to facial muscles suffered from self-injurious biting leading to injuries of the tongue, lip, and fingers (Figure 2 and Figure 3). All patients with self-injurious biting required BoNT to bilateral masseter (range of 80–110 units) and bilateral temporalis (range of 35–80 units) (Table 4). All patients receiving BoNT for self-injurious biting demonstrated meaningful benefit with no further injuries from biting during the duration of BoNT action (Table 3). Although dosing regimen was not planned using weight-based calculations, total units administered per kilogram for patients with self-injurious biting ranged from 10.9 to 31.7 U/kg (average 17.6 U/kg). There were no adverse effects and all patients received repeat injections approximately 3 months following initial injections. One patient required tooth extraction due to early wearing off (duration of effect approximately 8 weeks) despite escalating doses.

Six patients with Tourette syndrome underwent injections of neck muscles to include splenius capitis, scalene, and sternocleidomastoid (Table 3 and Table 4). These patients exhibited neck tics that were painful and quite forceful that, if left untreated, risked development of cervical myelopathy [20]. The SIB in these patients was caused chiefly by repeat neck extension (“whiplash”) tics. Three patients improved markedly with botulinum toxin injections into the neck muscles without any adverse effects. One patient with tics manifested by forceful arm supination received injections to the supinator muscle, which resulted in finger extensor weakness and no clear benefit. One patient was lost to follow-up.

**Figure 4:**
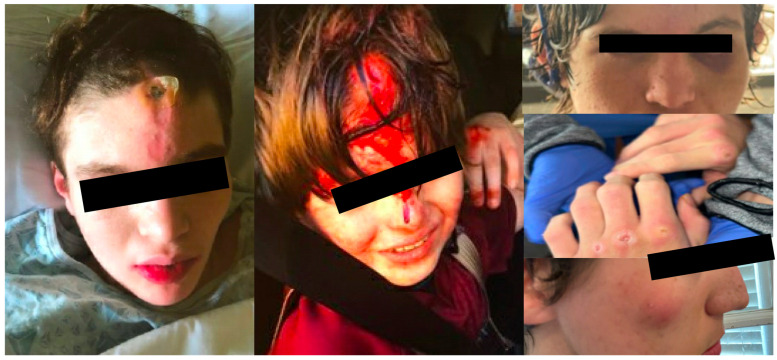
Injuries sustained by patients with hitting stereotypy prior to initiating botulinum toxin injections to include injuries to the face, scalp, and hands.

Three patients were treated with botulinum toxin injections for injuries sustained by complex motor stereotypy manifested by forceful hitting and punching (Table 3; Figure 4). Despite doses of up to 600 units (300 U in each bicep), there was no improvement in the SIB. There were no adverse effects in these patients, however, none had noted meaningful benefit. One patient with face-kicking stereotypy (Video S1) received BoNT injections to the iliopsoas, gluteus maximus, and adductor group, after which caregivers reported meaningful and sufficient benefit with no further injuries and no adverse effects.

## 3. Discussion

Symptoms of SIB range from mild (minor tissue damage with scratches or bruising) to severe (permanent tissue loss, blindness, deafness, or life-threatening consequences). Within movement disorders, SIB is seen most commonly in the setting of Tourette syndrome [1,21,22,23], but other genetic conditions, such as LNS and neuroacanthocytosis, have been also associated with SIB [5,24]. In patients with genetic causes of SIB, the behaviors themselves can be very frequent, occurring at least once every 30 min in 18% of the patients [5]. Rarely, SIB can also be seen in autoimmune conditions, such as anti-N-methyl-D-aspartate receptor encephalitis, or after exposure to certain drugs [1].

SIB can lead not only to permanent disability, but also can be potentially life-threatening [2,4,5,6]. Furthermore, the estimated cost of care associated with SIB in the United States in 1989 exceeded $3 billion (over $7 billion in today’s dollars), which, given the rise of healthcare costs, has no doubt escalated since then [25].

The pathophysiology underlying SIB is not well described and may differ depending on etiology. Theories implicate an interplay between three main factors: (1) Behavioral inflexibility or patterned/repetitive or compulsive behaviors, (2) intellectual disability, and (3) pathological reinforcement learning or abnormal neurotransmission, which may involve dopaminergic, opioid, or serotonergic pathways [1,3,4,6,24,25,26,27]. Of note, the behavioral inflexibility and patterned behaviors may not be restricted to the movements involved in the SIBs, such as tics and stereotypies, but may be manifested through obsessive compulsive behaviors or impulsivity [6,21,22]. Another theory involves the endogenous opioid system and control of noxious stimuli, in which acute pain results in activation of inhibitory pathways within the endogenous opioid system and plays a role in alleviating chronic pain [25,28]. The latter has particular implications for the course of SIB [4] as delay in treatment may lead to physiological changes contributing to a chronic pain state through nociceptive changes [25]. These changes then create a feed forward process which would make treatment even more difficult. Therefore, early and effective treatment is critical not only to prevent acute injury, but also to mitigate against chronic disability.

Numerous treatments of SIB are available, although none have been systematically reviewed. Treatments range from less invasive, such as physical restraints and behavioral therapy, to pharmacotherapy, including alpha-2 agonists, benzodiazepines, antipsychotics, VMAT-2 inhibitors, muscle relaxants, SSRI/antagonists, mood stabilizers, anxiolytics, and naltrexone, to more invasive interventions, including tooth extraction and deep brain stimulation. We describe the first series of patients with SIB treated with BoNT injections.

Our series of patients provides evidence that there is a role for BoNT injections as part of a multimodal, tiered approach in the management of SIB. Although patients with SIB involving the upper extremity had no clear benefit from BoNT injections, this may have been due to underdosing or inappropriate selection of muscles, as there were no noted adverse effects apart from one patient who noted finger extensor weakness when supinator was injected. In contrast, all patients with self-injurious biting that underwent BoNT targeting the bilateral masseter and temporalis muscles had meaningful benefit in the SIB; none of these patients experienced adverse effects. Half of the patients with forceful neck extension tics had meaningful benefit with BoNT targeting the neck muscles, and none experienced adverse effects. “Whiplash” tics must be viewed as a medical emergency, as there are several reports in the literature of quadriparesis or quadriplegia resulting from tic-related compressive cervical myelopathy that, fortunately, resolved after timely BoNT injections into the neck muscles [20,29,30]. 

BoNT acts by inhibiting the release of acetylcholine from the presynaptic nerve terminal, and thus interfering with fusion of the synaptic vesicle with the presynaptic plasma membrane [31,32]. This mechanism is known to relax the muscle; however, recent studies have provided evidence that BoNT may also be effective in the treatment of neuropathic pain [32,33,34]. The clinical rationale for the use of BoNT in our patients was to lessen the intensity of the movements, and thus reduce the risk of further injury from repetitive behaviors.

Limitations of this study include its retrospective, open-label design and relatively small sample size. Injections were performed using anatomic landmarks, without ultrasound or electromyographic guidance. Although this study was not designed to compare different techniques of administration of BoNT, we believe that the site of needle insertion is based on the clinical observation of dynamic positioning during the abnormal movement, surface anatomy, palpation, local muscles mass, range of motion, and other clinical factors. These clinical assessments are critical to the appropriate selection of the target muscles. There are no well-designed, controlled, studies that have demonstrated superiority of electromyography guidance or electrical stimulation or ultrasound-guided injection over the use of anatomical landmarks [35,36]. Indeed, a recent study showed no difference between ultrasound and electrical stimulation in the outcomes of BoNT treatment of focal hand dystonia and upper limb spasticity [37]. Finally, there are more than 100 instruments available for use in measuring self-harming behaviors, although most are not validated, and none are validated for use in pediatric patients with intellectual disability [38]. Therefore, we relied on reports from the caregivers and clinical documentation.

## 4. Conclusions

This study, based on a retrospective review of patients with SIB refractory to multiple therapeutic interventions, provides evidence that BoNT is a useful therapeutic modality in some patients. Despite the limitations of our study, the observations support the conclusion that BoNT can provide meaningful benefit in self-injurious biting and “whiplash” tics, therefore preventing serious and permanent disability. 

## 5. Materials and Methods

Here, we describe 17 patients presenting to our pediatric movement disorders clinic for management of SIB refractory to pharmacologic and mechanical interventions and treated with BoNT injections (onabotulinumtoxinA) as part of clinical care by child neurologists trained in movement disorders. Patients were followed clinically, and we discussed features, dosing regimen, and outcomes through a review of clinical documentation and caregiver report and completed retrospectively. During typical follow-up visits, full examinations were performed to include the evaluation of any repeated injuries as well as observation of the SIB (if still present). Careful history was also obtained to include the caregiver’s report of frequency and severity of SIB and recurrence of injury from SIB. Caregivers were also questioned regarding latency of BoNT effects, degree, and duration of effect, and any adverse reactions related to the treatment. Finally, caregivers were asked to compare the severity of SIB while BoNT is in effect to the patient’s level of SIB prior to treatment. Patients received repeat injections every 12 weeks. Response to BoNT was determined based on chart review and patient and caregiver response to the survey based on a 1- to 4-point Likert scale (1 = normal or mildly ill, 2 = moderately ill, 3 = markedly ill, and 4 = severely ill), comparing illness severity before and after BoNT injections (Table 2). 

## Figures and Tables

**Table 1:** Summary of patient demographics.

Characteristics	Number of Patients (%)
Sex	
Male	11 (65)
Female	6 (35)
Age	
11–35 mo	4 (23.5)
3–7 yr	1 (5.9)
8–12 yr	3 (17.6)
13–20 yr	9 (52.9)
**Comorbid Diagnoses ^1^**	
Intellectual disability/Global developmental delay	10 (58.8)
Tourette syndrome/Chronic motor tic disorder	4 (23.5)
Autism spectrum disorder	3 (17.6)
None of the above	1 (5.9)

^1^ One patient carried a diagnosis of intellectual disability and autism spectrum disorder.

**Table 2:** Characteristics of patients with SIB and prior treatments.

Patient	Sex	Age	Phenomenology	Comorbid Diagnosis/Etiology	Prior Treatments	BoNT Outcome
1	M	16 mo	Self-injurious biting	Global developmental delay/congenital insensitivity to pain with anhidrosis	Tooth extraction Physical restraints	1
2	F	16 yr	Whiplash neck tics	Tourette syndrome	None	Unknown ^a^
3	F	18 yr	Hitting/punching stereotypy	Intellectual disability/Aicardi syndrome	Physical restraints Alpha-2 agonists Benzodiazepines	4
4	M	15 yr	Forceful arm supination tics	Tourette syndrome	Antipsychotics VMAT-2 inhibitors	4 ^b^
5	F	11 yr	Self-injurious biting	Intellectual disability/cardiofaciocutaneous syndrome	Antipsychotics	1
6	M	13 yr	Whiplash neck tics	Chronic motor tic disorder	Antipsychotics Muscle relaxants	1
7	M	16 yr	Hitting/punching stereotypy	Intellectual disability	Physical restraints Antipsychotics SSRI/antagonists	4
8	F	17 yr	Whiplash neck tics	Intellectual disability	None	1
9	M	11 mo	Self-injurious biting	Recurrent nocturnal tongue biting	Benzodiazepines	1
10	M	5 yr	Self-injurious biting	Intellectual disability	Physical restraints VMAT-2 inhibitors Alpha-2 agonists	1
11	M	23 mo	Self-injurious biting	Global developmental delay/Lesch-Nyhan syndrome	Physical restraints Benzodiazepines	1 ^c^
12	F	22 mo	Self-injurious biting	Global developmental delay/perinatal tuberculosis meningitis with ischemic stroke	Teeth extraction Physical restraints Benzodiazepines	1
13	M	17 yr	Hitting/punching stereotypy	Intellectual disability and autism spectrum disorder	Physical restraints SSRI/antagonists Mood stabilizers Antipsychotics Benzodiazepines VMAT-2 inhibitors Alpha-2 agonists	4
14	F	15 yr	Whiplash neck tics	Autism spectrum disorder	Mood stabilizers Alpha-2 agonists SSRI/antagonists	1
15	M	10 yr	Whiplash neck tics	Autism spectrum disorder/creatine transporter deficiency	Antipsychotics Alpha-2 agonists Naltrexone SSRI/antagonists	4
16	M	11 yr	Kicking stereotypy	Intellectual disability/mitochondrial disorder	Physical restraints VMAT-2 inhibitors Benzodiazepines	1 ^c^
17	M	18 yr	Whiplash neck tics	Tourette syndrome	Behavioral therapy Antipsychotics VMAT-2 inhibitors Mood stabilizers	4

M = Male, F = Female. VMAT-2 = Vesicular Monoamine Transporter 2. SSRI = Selective serotonin reuptake inhibitor. ^a^ Patient was lost to follow-up. ^b^ Experienced finger extensor weakness. ^c^ Short duration of benefit.

**Table 3:** Phenomenology of self-injurious behaviors and response to botulinum neurotoxin injections.

Phenomenology	Body Region Injected	Number of Patients (%)	Meaningful Benefit (%)	Duration of Benefit (Weeks) ^1^
Self-injurious Biting	Face	6 (35.3)	6 (100)	6–12
Tics *Whiplash neck extension tics* *Forceful arm supination*	Neck Upper Extremity	7 (41.2) 6 1	3 ^1^ (42.9) 3 ^2^ 0	8–16
Stereotypy *Hitting/Punching* *Kicking*	Upper Extremity Lower Extremity	4 (23.5) 3 1	1 (25) 0 1	8

^1^ Duration of benefit for those with meaningful benefit. ^2^ One patient was lost to follow-up.

**Table 4:** Final dosing regimen used in treatment of self-injurious behaviors.

Body Region	Muscles Injected	Typical Dose per Muscle (Mean) (U)	Dose Variability (Standard Deviation) (U)	Dose Limits per Muscle (Min–Max) (U)
Face	Masseter	88.3	13.3	80–110
	Temporalis	57.5	15.1	35–80
Neck	Splenius capitis	65	4.1	60–70
	Scalene	63.8	37.7	40–120
	Sternocleidomastoid	40	10	30–50
Upper Extremity	Biceps	300	100	200–400
	Pectoralis major *	100	N/A	N/A
	Latissimus dorsi *	200	N/A	N/A
	Supinator *	40	N/A	N/A
Lower Extremity	Iliopsoas *	200	N/A	N/A
	Gluteus maximus *	300	N/A	N/A
	Adductor *	200	N/A	N/A

* Doses provided are those of single patients; therefore, no standard deviations or ranges are provided. U = units.

## Data Availability

The data presented in this study are available on request from the corresponding author. The data are not publicly available due to patient privacy reasons.

## Supplementary Materials

The following supporting information can be downloaded at: https://www.mdpi.com/article/10.3390/toxins15040236/s1. Video S1: Self injurious stereotypy with repetitive kicking of the head prior to BoNT.

## References

[1] Fischer J.-F., Mainka T., Worbe Y., Pringsheim T., Bhatia K., Ganos C. (2020). Self-injurious behaviour in movement disorders: Systematic review. J. Neurol. Neurosurg. Psychiatry.

[2] Denis J., Noortgate W.V.D., Maes B. (2011). Self-injurious behavior in people with profound intellectual disabilities: A meta-analysis of single-case studies. Res. Dev. Disabil..

[3] Schroeder S.R., Oster-Granite M.L., Berkson G., Bodfish J.W., Breese G.R., Cataldo M.F., Wong D.F. (2001). Self-injurious behavior: Gene–brain–behavior relationships. Ment. Retard. Dev. Disabil. Res. Rev..

[4] Dimian A.F., Symons F.J. (2022). A systematic review of risk for the development and persistence of self-injurious behavior in intellectual and developmental disabilities. Clin. Psychol. Rev..

[5] Huisman S., Mulder P., Kuijk J., Kerstholt M., van Eeghen A., Leenders A., van Balkom I., Oliver C., Piening S., Hennekam R. (2018). Self-injurious behavior. Neurosci. Biobehav. Rev..

[6] Davies L., Oliver C. (2016). Self-injury, aggression and destruction in children with severe intellectual disability: Incidence, persistence and novel, predictive behavioural risk markers. Res. Dev. Disabil..

[7] Sabus A., Feinstein J., Romani P., Goldson E., Blackmer A. (2019). Management of Self-Injurious Behaviors in Children with Neurodevelopmental Disorders: A Pharmacotherapy Overview. Pharmacotherapy.

[8] Russell P.S.S. (2006). Self-Injurious Behavior to the Lower Extremities among Children with Atypical Development: A Diagnostic and Treatment Algorithm. Int. J. Low. Extrem. Wounds.

[9] Tambirajoo K., Furlanetti L., Hasegawa H., Raslan A., Gimeno H., Lin J.P., Selway R., Ashkan K. (2021). Deep Brain Stimulation of the Internal Pallidum in Lesch–Nyhan Syndrome: Clinical Outcomes and Connectivity Analysis. Neuromodulation.

[10] Anandan C., Jankovic J. (2021). Botulinum Toxin in Movement Disorders: An Update. Toxins.

[11] Jankovic J. (2018). An Update on New and Unique Uses of Botulinum Toxin in Movement Disorders. Toxicon.

[12] Andraweera N.D., Andraweera P.H., Lassi Z.S., Kochiyil V. (2020). Effectiveness of Botulinum Toxin A Injection in Managing Mobility-Related Outcomes in Adult Patients With Cerebral Palsy. Am. J. Phys. Med. Rehabil..

[13] Moretti A. (2020). Is botulinum toxin effective and safe for motor and phonic tics in patients affected by Tourette syndrome? A Cochrane Review summary with commentary. Dev. Med. Child Neurol..

[14] Kwak C.H., Hanna P.A., Jankovic J. (2000). Botulinum Toxin in the Treatment of Tics. Arch. Neurol..

[15] Karian V., Yu-Hsing Chang C., Schefter Z.J., Lebel A. (2022). Can Botox Reduce Self-Injurious Behavior in Young Patients with Chronic Head Pain and Autism/Developmental Delay?. Autism. Open Access.

[16] Gutierrez C., Pellene A., Micheli F. (2008). Botulinum Toxin: Treatment of Self-Mutilation in Patients with Lesch-Nyhan Syndrome. Clin. Neuropharmacol..

[17] Dressler D., Altavista M.C., Altenmueller E., Bhidayasiri R., Bohlega S., Chana P., Chung T.M., Colosimo C., Fheodoroff K., Garcia-Ruiz P.J. (2021). Consensus guidelines for botulinum toxin therapy: General algorithms and dosing tables for dystonia and spasticity. J. Neural Transm..

[18] Perotto A.O. (2011). Anatomical Guide for the Electromyographer: The Limbs and Trunk.

[19] Jost W. (2019). Atlas of Botulinum Toxin Injection: Dosage, Localization, Application.

[20] Baizabal-Carvallo J.F., Alonso-Juarez M., Jankovic J. (2022). Self-injurious behavior in Tourette syndrome. J. Neurol..

[21] Cheung M.Y.C., Shahed J., Jankovic J. (2007). Malignant Tourette Syndrome. Mov. Disord..

[22] A Mathews C., Waller J., Glidden D., Lowe T.L., Herrera L.D., Budman C.L., Erenberg G., Naarden A., Bruun R.D., Freimer N.B. (2004). Self injurious behaviour in Tourette syndrome: Correlates with impulsivity and impulse control. J. Neurol. Neurosurg. Psychiatry.

[23] Claes L., Vandereycken W. (2007). Self-injurious behavior: Differential diagnosis and functional differentiation. Compr. Psychiatry.

[24] Arron K., Oliver C., Moss J., Berg K., Burbidge C. (2011). The Prevalence and Phenomenology of Self-Injurious and Aggressive Behaviour in Genetic Syndromes. J. Intellect. Disabil. Res..

[25] Symons F.J. (2011). Self-injurious behavior in neurodevelopmental disorders: Relevance of nociceptive and immune mechanisms. Neurosci. Biobehav. Rev..

[26] Oliver C., Petty J., Ruddick L., Bacarese-Hamilton M., Oliver C. (2012). The Association Between Repetitive, Self-Injurious and Aggressive Behavior in Children with Severe Intellectual Disability. J. Autism Dev. Disord..

[27] Richman D.M., Barnard-Brak L., Bosch A., Thompson S., Grubb L., Abby L. (2013). Predictors of self-injurious behaviour exhibited by individuals with autism spectrum disorder. J. Intellect. Disabil. Res..

[28] Peebles K.A., Price T.J. (2012). Self-Injurious Behaviour in Intellectual Disability Syndromes: Evidence for Aberrant Pain Signalling as a Contributing Factor. J. Intellect. Disabil. Res..

[29] Patterson A.L., Choudhri A.F., Igarashi M., McVicar K., Shah N., Morgan R. (2016). Severe Neurological Complications Associated with Tourette Syndrome. Pediatr. Neurol..

[30] Adler C.H., Zimmerman R.S., Lyons M.K., Simeone F., Brin M.F. (1996). Perioperative Use of Botulinum Toxin for Movement Disorder-Induced Cervical Spine Disease. Mov. Disord..

[31] Jankovic J. (2017). Botulinum Toxin: State of the Art. Mov. Disord..

[32] Lippi L., de Sire A., Folli A., D’abrosca F., Grana E., Baricich A., Carda S., Invernizzi M. (2022). Multidimensional Effectiveness of Botulinum Toxin in Neuropathic Pain: A Systematic Review of Randomized Clinical Trials. Toxins.

[33] Zhou K., Luo W., Liu T., Ni Y., Qin Z. (2022). Neurotoxins Acting at Synaptic Sites: A Brief Review on Mechanisms and Clinical Applications. Toxins.

[34] Attal N., de Andrade D.C., Adam F., Ranoux D., Teixeira M.J., Galhardoni R., Raicher I., Üçeyler N., Sommer C., Bouhassira D. (2016). Safety and Efficacy of Repeated Injections of Botulinum Toxin A in Peripheral Neuropathic Pain (BOTNEP): A Randomised, Double-Blind, Placebo-Controlled Trial. Lancet Neurol.

[35] Semerdjieva N.I. (2016). How Useful Are Localization Techniques in Botulinum Toxin Injections for Dystonia and Spasticity Indications?. Botulinum Toxin Therapy Manual for Dystonia and Spasticity.

[36] Lim E.C., Quek A.M., Seet R.C. (2011). Accurate targeting of botulinum toxin injections: How to and why. Park. Relat. Disord..

[37] Lungu C., Nmashie A., George M.C., Karp B.I., Alter K., Shin S., Tse W., Frucht S.J., Wu T., Koo V. (2022). Comparison of Ultrasound and Electrical Stimulation Guidance for Onabotulinum Toxin-A Injections: A Randomized Crossover Study. Mov. Disord. Clin. Pract..

[38] Faura-Garcia J., Orue I., Calvete E. (2021). Clinical assessment of non-suicidal self-injury: A systematic review of instruments. Clin. Psychol. Psychother..

